# Non-COVID-19 patients in times of pandemic: Emergency department visits, hospitalizations and cause-specific mortality in Northern Italy

**DOI:** 10.1371/journal.pone.0248995

**Published:** 2021-03-22

**Authors:** Luca Santi, Davide Golinelli, Andrea Tampieri, Gabriele Farina, Manfredi Greco, Simona Rosa, Michelle Beleffi, Bianca Biavati, Francesca Campinoti, Stefania Guerrini, Rodolfo Ferrari, Paola Rucci, Maria Pia Fantini, Fabrizio Giostra

**Affiliations:** 1 Department of Emergency, Medicina d’Urgenza e Pronto Soccorso, Policlinico S. Orsola-Malpighi, Bologna, Italy; 2 Department of Biomedical and Neuromotor Sciences (DIBINEM), Alma Mater Studiorum - University of Bologna, Bologna, Italy; 3 Department of Emergency, Medicina d’Urgenza e Pronto Soccorso. Ospedale S. Maria della Scaletta, Imola, Italy; 4 Emergency Medicine Specialization School, Alma Mater Studiorum - Università di Bologna, Bologna, Italy; Universita degli Studi di Ferrara, ITALY

## Abstract

The COVID-19 pandemic forced healthcare services organization to adjust to mutating healthcare needs. Not exhaustive data are available on the consequences of this on non-COVID-19 patients. The aim of this study was to assess the impact of the pandemic on non-COVID-19 patients living in a one-million inhabitants’ area in Northern Italy (Bologna Metropolitan Area-BMA), analyzing time trends of Emergency Department (ED) visits, hospitalizations and mortality. We conducted a retrospective observational study using data extracted from BMA healthcare informative systems. Weekly trends of ED visits, hospitalizations, in- and out-of-hospital, all-cause and cause-specific mortality between December 1^st^, 2019 to May 31^st^, 2020, were compared with those of the same period of the previous year. Non-COVID-19 ED visits and hospitalizations showed a stable trend until the first Italian case of COVID-19 has been recorded, on February 19^th^, 2020, when they dropped simultaneously. The reduction of ED visits was observed in all age groups and across all severity and diagnosis groups. In the lockdown period a significant increase was found in overall out-of-hospital mortality (43.2%) and cause-specific out-of-hospital mortality related to neoplasms (76.7%), endocrine, nutritional and metabolic (79.5%) as well as cardiovascular (32.7%) diseases. The pandemic caused a sudden drop of ED visits and hospitalizations of non-COVID-19 patients during the lockdown period, and a concurrent increase in out-of-hospital mortality mainly driven by deaths for neoplasms, cardiovascular and endocrine diseases. As recurrencies of the COVID-19 pandemic are underway, the scenario described in this study might be useful to understand both the population reaction and the healthcare system response at the early phases of the pandemic in terms of reduced demand of care and systems capability in intercepting it.

## Introduction

Globally, the Coronavirus Disease (COVID-19) pandemic represents a dramatic burden for healthcare services. Italy was the first Western country affected by the pandemic. The first case of local transmission of Severe Acute Respiratory Syndrome Coronavirus 2 (SARS-CoV-2) in Italy was confirmed in a thirty eight-year-old man in the municipality of Codogno (Lombardy region) on February 19^th^, 2020 [1], while on February 21^st^, a resident of Vo’, a small town near Padua (Veneto Region), died of pneumonia due to SARS-CoV-2 infection. [2, 3] In the following weeks, an exponential growth in the number of cases and deaths in the neighboring regions of northern Italy was observed. As a consequence, the national government enforced as containment measures a complete country lockdown on March 10^th^, 2020: at that stage the confirmed cases in Italy were 10,149 and 631 deaths [4]. Hospitals and Emergency Departments (EDs) were forced to rapidly adjust to this completely new situation in order to manage an extraordinarily high number of contagious patients with respiratory symptoms [5].

However, something else changed due to the pandemic as rapidly as the spread of SARS-CoV-2. As a matter of fact, the pandemic determined a sudden and significant reduction of non-COVID-19 patients seeking for treatment due to urgent medical conditions [6]. This reduction in ED visits might impact the health status of non-COVID patients with acute and chronic diseases [7].

In order to better understand this phenomenon and its possible consequences, we retrospectively analyzed amount and type of ED visits, hospitalizations and mortality during lockdown and the preceding and following periods, in the metropolitan area of Bologna (BMA) (~1,000,000 inhabitants), which is the principal town of one of the first and most affected region of Italy and Europe, Emilia Romagna.

The first aim of this study was to compare time trends of ED visits, hospitalizations, in- and out-of-hospital all-cause and cause-specific mortality of non-COVID-19 patients during the first months of the pandemic with the same period of 2019.

The secondary aim was to determine whether the reason for ED access changed between 2019 and 2020, and whether the pattern of cause-specific mortality and the setting of death changed between the two years.

We first hypothesized that trends of ED visits might have been influenced by age, as school closure and social distancing measures might have differently impacted on young and elderly health needs. The fear of contagion might also have differently affected the severity pattern of ED accesses. Indeed, we predicted a significant reduction of less severe conditions referring to EDs, whereas no decrease of emergency conditions was expected, including the need of urgent surgical intervention or ICU treatment. No remarkable changes in all-cause and cause-specific mortality, and in the place of death were hypothesized.

## Materials and methods

### Study population

We conducted a retrospective observational study using anonymous aggregated data extracted from the healthcare administrative databases and information systems of BMA, Northern Italy.

The study population consisted of all residents in BMA, which encompasses the city of Bologna and the neighboring municipalities, for a total of 1,019,875 citizens as of January 1^st^, 2020 [8]. The demographic characteristics of the population are reported in S1 Table in S1 File.

In the BMA, the first patient reported with SARS-CoV-2 infection was recorded on February 28^th^. On May 31^st^, the number of infected patients was 5,021 with 684 deaths [4].

The BMA healthcare system consists of twelve hospitals equipped with EDs. Among these, four are located in the urban area and two of these are large university hospitals, four are suburban whereas the last four are rural hospitals. The volume of patients annually assessed in the different EDs ranges from ~3,000 up to ~150,000 in 2019.

This study follows the STROBE reporting guidelines for observational studies.

### Study period and data

We examined the weekly trends of ED visits, hospitalizations of patients admitted from ED and in- and out-of-hospital all-cause and cause-specific mortality between December 1^st^, 2019 to May 31^st^, 2020, and compared them with the trends of the same period of the previous year.

The study period was subdivided into four periods, starting from the beginning of the flu peak up to four weeks after the end of the national lockdown. Namely, the following are the four subgroups of the study period:

- flu period (from December 1^st^, 2019 to February 23^rd^, 2020 the outbreak of the COVID-19 pandemic to the first containment measures, i.e. from the beginning of the flu period to week 49^th^ 2019 to week 8^th^ 2020);
- pre-lockdown period (from the initial alarm period in the absence of containment institutional measures until the lockdown institution, i.e. February 24^th^, 2020 to March 9^th^, 2020, weeks 9^th^ to 10^th^ 2020);
- lockdown period (March 10^th^, 2020 to May 3^rd^, 2020, weeks 11^th^ to 18^th^ 2020);
- post-lockdown period (i.e. the reopening period, from the May 4^th^, 2020 to May 31^st^, 2020, week 19^th^ to 22^nd^ 2020).

The data sources include the Emergency Department Database, the mortality registry updated to June 2020 and the Civil Protection Database [4]. For the purpose of the present study, the data extracted from the different databases were the following: weekly number of ED visits, type of ED visits (main diagnosis, severity code), patients characteristics (age, gender), weekly number of hospitalizations, department of hospitalization (medical, surgical, ICU), weekly number of deaths, cause of death (main diagnosis) and place of death (in-hospital, out-of-hospital).

ED visits are categorized by severity codes according to the triage protocols in four levels of urgency: white code (not-urgent condition), green code (postponable conditions); yellow code (medical condition requiring urgent care with no vital signs impairment but at-risk for deterioration); red code (medical condition with acute impairment of vital signs requiring emergency care) [9, 10].

The main diagnosis of ED visits and deaths was coded through the International Classification of Diseases Clinical Modification Ninth (ICD-9-CM) and Tenth (ICD-10-CM) versions, respectively (see in S1 File).

Non-COVID-19 cases were identified by excluding the suspected or confirmed COVID-19 cases (ICD-9-CM codes 480, 480.3, 079.82, V01.82, V01.79), in primary or secondary diagnosis of hospital discharge records. For all mortality data, the specific codes related to COVID-19 and SARS-CoV-2 infection (U00-U85) were ruled out.

### Statistical analysis

The demographic and clinical characteristics of the population were summarized using mean and standard deviation or absolute and percentage frequencies.

We analyzed and displayed trends of ED visits, hospital admissions, and mortality, divided into the four time periods and compared them with those of the same periods of the previous year.

We carried out a preliminary analysis comparing the time trend of ED visits, hospitalizations and mortality rates in 2019 with those of the years 2014–2018. This analysis showed no significant changes in ED visits, hospitalizations and mortality over time. Therefore, we considered 2019 as the reference year.

The trends of ED visits, hospitalizations and all-cause mortality were analyzed using the Joinpoint Trend Analysis Software 4.8.0.1 (Statistical Research and Applications Branch, National Cancer Institute, USA) [9]. Joinpoint models were used to identify changes in the slopes of ED visits and hospitalizations during the 26 weeks of observation and to compare also the trends of 2 years. Specifically, this software starts by fitting trend data with a 0 joinpoint model, which is a straight line, and then tests whether more joinpoints are statistically significant and must be added to the model. We modeled the weekly counts of events (the dependent variable) as a function of the week using a Poisson model of variation. The significance of the percentage rate changes within the 26 week periods was tested using a Monte Carlo Permutation method [11]. The joinpoint regressions of mortality were compared between the two periods using the comparability test, that tests whether two joinpoint regression functions are parallel (test of parallelism) [12]. When the null hypothesis of parallelism is rejected, there is an indication that regression curves change their slope at different time points during the observation period.

For what concerns the age of non-COVID-19 ED patients during the pandemic, it was classified into three groups: pediatric patients (0–14 years), adults (15–64 year) and elderly (≥ 65 years). We also classified ED visits according to the triage code adopted by the BMA EDs (red, yellow, green and white) and by ICD-9-CM disease category and specific cause of death. We also analyzed the trends of all-cause, cause-specific and in-hospital (public or private) and out-of-hospital (home, social welfare structure, other) mortality for the years 2019 and 2020. The latter was also split into cause-specific out-of-hospital mortality.

Incidence rate ratios (IRRs) of hospitalizations and mortality were obtained by dividing the counts of events for the year 2020 by the counts of events for the year 2019, under the assumption that the population at risk was the same in both time periods. No offset was used to obtain our estimates, because the exposure was the same in the two study periods (26 weeks). Ninety-five percent confidence intervals were calculated under the assumption of normality for the sampling distribution of ln(IRR).

Statistical analyses were carried out using IBM SPSS, version 25.0.

### Ethical approval

This study has been approved by the Emilia Romagna AVEC research ethics committee board with identifier n° 726/2020/Oss/AOUBo on 03.08.2020. This retrospective study was carried out in conformity with the regulations on data management with the Italian law on privacy (Legislation Decree 196/2003 amended by Legislation Decree 101/2018). The datasets generated and/or analysed during the current study are property of Bologna Metropolitan areas healthcare authorities (AUSL Bologna, AUSL Imola), and, although anonymized, they are not publicly available due to the current regulation on privacy. Data were pseudonymized prior to the analysis and each patient was assigned a unique identifier that does not allow to trace the patient’s identity or other sensitive data. Pseudonymized administrative data can be used without a specific written informed consent when patient information is collected for healthcare management and healthcare quality evaluation and improvement (according to art. 110 on medical and biomedical and epidemiological research, Legislation Decree 101/2018). The database used in this study includes the following aggregated information: date of ED visit, primary and secondary diagnosis, triage code, gender, age (grouped into classes), destination at ED discharge, date of death, cause of death, setting of death. Patients and the public were not involved in the design or planning of the study. All procedures performed in this study were in accordance with the 1964 Helsinki Declaration and its later amendments.

## Results

**Fig 1:**
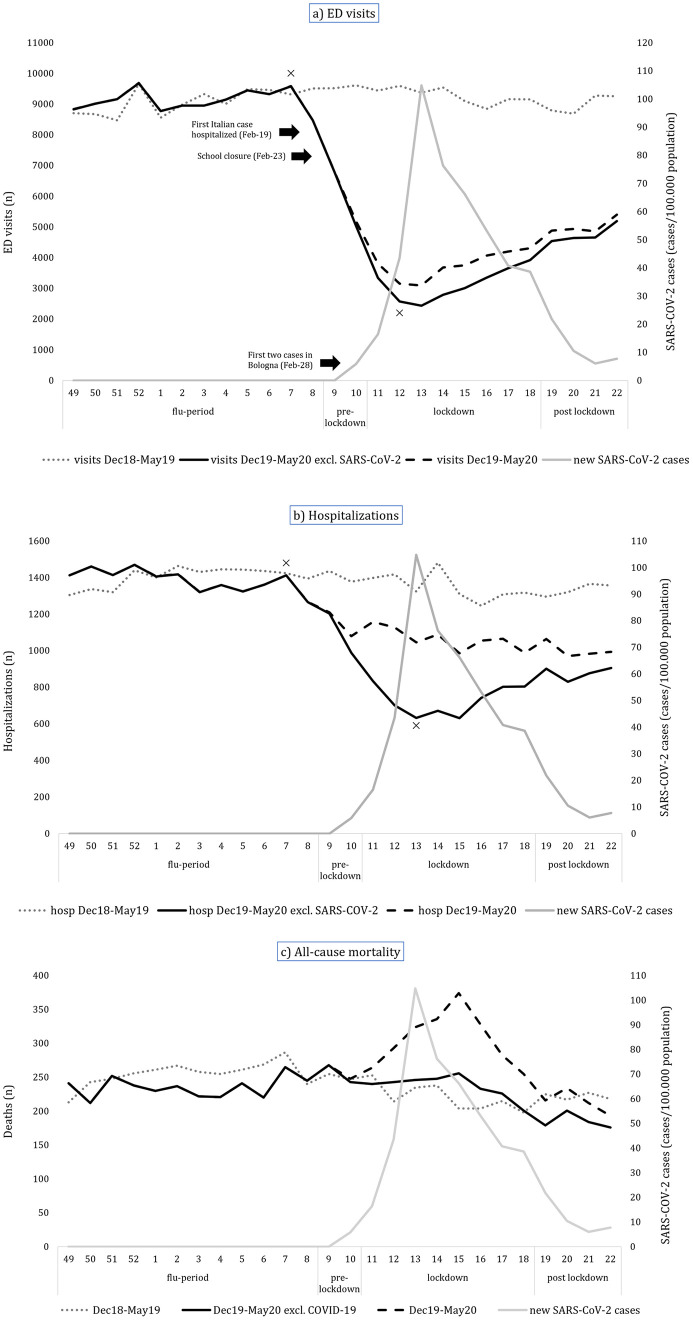
Number of non-COVID-19 ED visits (a), hospitalizations (b) and deaths (c) during the study periods for the years 2019 and 2020. For comparison, we displayed also the incidence of COVID-19 cases and the overall number of ED visits, hospitalizations and deaths.

The empirical trends of ED visits, hospitalizations and mortality in 2019 and 2020 for non-COVID-19 patients are reported in Fig 1.

### ED visits

Fig 1a shows that the number of non-COVID-19 ED visits was almost stable in the year 2019 until the end of 2020 flu period, then dropped simultaneously with the exponential growth of the SARS-CoV-2 case curve. Afterwards, during the lockdown period the number of ED visits started to rise, but with a slower pace when compared to the previous decrease.

Using joinpoint analysis, consistent with the empirical trend, the hypothesis of parallelism of the two regression curves was rejected and two changes in slope were identified in 2020: the first was a significant (p<0.05) reduction in ED visits starting from week nine (pre-lockdown period), leading to the lowest numbers at the thirteenth week during lockdown and the second was a significant increase (p<0.05) at the fourteenth week (see S1 Fig in S1 File). During the lockdown period the overall reduction in ED visits compared to same period of the previous year was -66.2% (S2 Table in S1 File). ED accesses’ decrease occurred during the steep climb of COVID-19 spread until the peak. The subsequent slow increase in ED visits never returned to the previous year levels, not even at the end of the study period, four weeks after the end of the national lockdown.

The reduction in the number of ED visits was found in all age groups, throughout all study periods (except for the flu period) (S3 Table in S1 File). The highest decrease was observed in pediatric patients during lockdown (-83.2%), although even adults and elderly patients ED accesses declined by 62.9% and 64.0%, respectively (S3 Table in S1 File).

**Fig 2:**
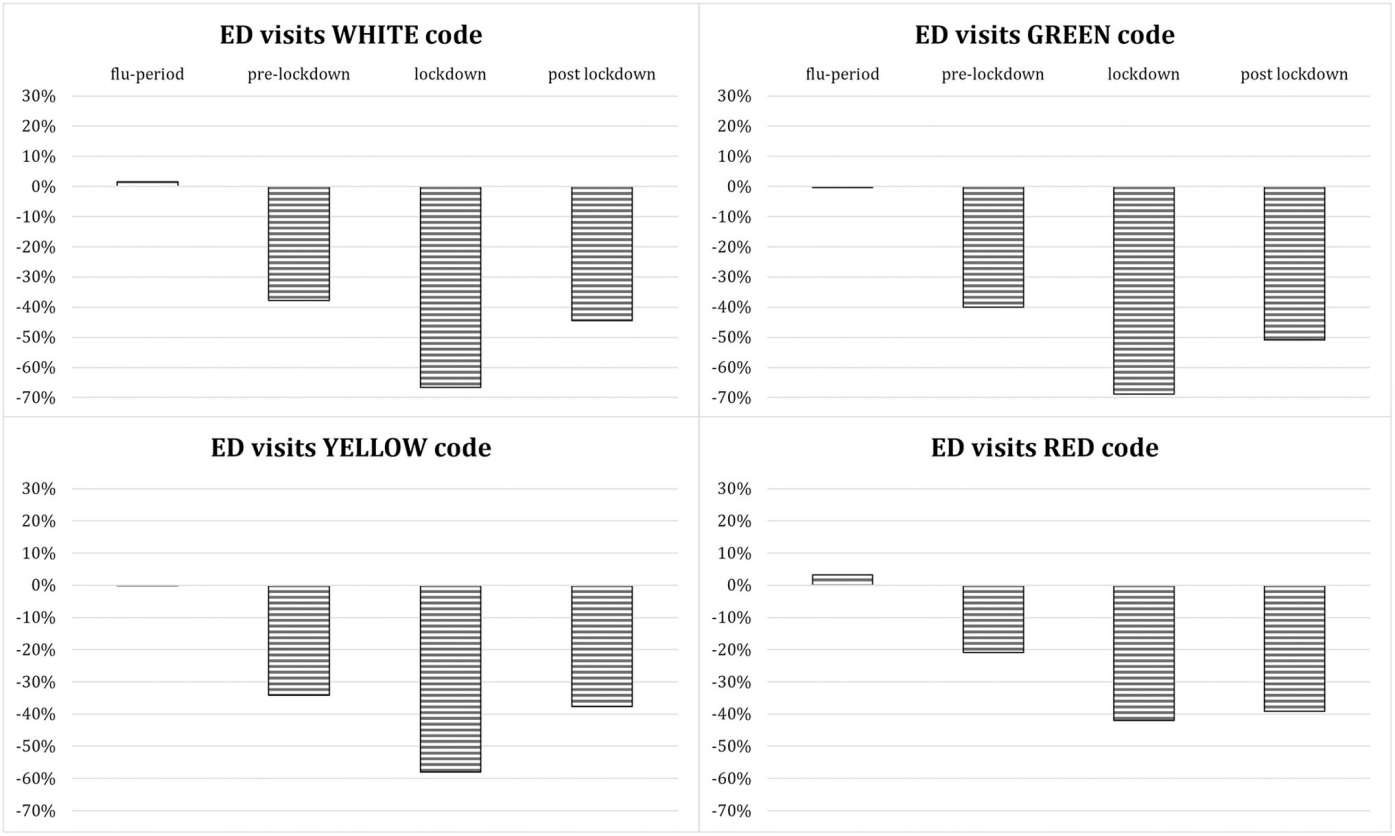
Percentage change in ED visits by severity code compared to the previous year.

For what concerns triage severity codes of non-COVID-19 patients, a reduction regarded similarly the four triage codes (Fig 2) during the study periods (except for the flu period) if compared to the previous year, with the most important decrease (between 60% and 70%) observed during lockdown.

**Fig 3:**
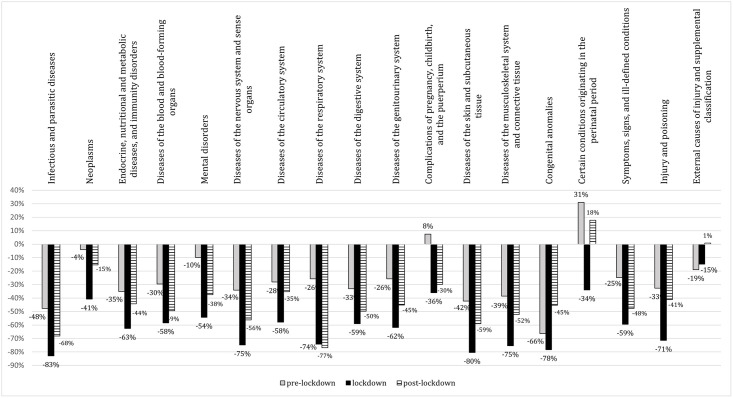
Percentage change in ED visits by disease category compared to the previous year.

As to non-COVID ED visits for specific conditions, our analysis demonstrated a reduction which broadly affected all disease categories, ranging from 41% to 83% in the lockdown period (Fig 3).

The decrease of ED visits was more pronounced for infectious and parasitic diseases (-83%), skin and subcutaneous tissue pathogenic conditions (-80%), and congenital anomalies (-78%) (Fig 3). A remarkable decrease in ED visits was also observed for conditions usually significantly impacting on BMA’s EDs [13], such as diseases of the cardiovascular system (-58%) as well as endocrine, nutritional and metabolic diseases (-63%). Specifically, the remarkable reduction in ED visits interested life-threatening illnesses, such as ischemic heart disease (-49.7%), intracranial hemorrhages (-33.3%) and ischemic cerebrovascular disorders (-45.8%) (S4 Table in S1 File). Lower reductions or a modest increase were recorded in complications of pregnancy, childbirth and the puerperium and some morbid conditions of perinatal origin (+8% during pre-lockdown, -36% during lockdown, and -30% during post-lockdown period) (Fig 3).

### Hospitalizations

Fig 1b shows hospitalizations trends in the study periods, which appears to be quite stable during year 2019 until the end of 2020 flu period. Similarly to ED visits trends, hospital admissions started to drop simultaneously with the exponential growth of the local SARS-CoV-2 case curve. The number of hospitalizations started to rise again during the lockdown period, but with a slower pace.

Joinpoint analysis showed that in 2019 hospitalizations slightly increased up to week 2 and then declined, while in 2020 non-COVID hospitalizations declined slightly but significantly between weeks 49 and 8, then more steeply between weeks 8 and 13 before the peak of incidence of COVID-19 cases, and moderately increased afterwards (see S2 Fig in S1 File).

The overall reduction of hospitalizations was -46·1% during the lockdown period compared to previous year (S2 Table in S1 File). Admissions to all hospital departments declined during and after lockdown (Table 1). During lockdown, admissions reduction was significant and interested likewise surgical and medical departments (-36.0%) as well as ICUs (-36.4%).

**Table 1:** Number and percentage change of hospitalizations in medicine, surgery and ICU departments, year 2020 compared to 2019.

	2020 (n)				Change from previous year (%)				Incidence Rate Ratio [95% CI]			
	Flu-period	Pre-lockdown	Lockdown	Post-lockdown	Flu-period	Pre-lockdown	Lockdown	Post-lockdown	Flu-period	Pre-lockdown	Lockdown	Post-lockdown
**Medicine**	9,903	1,493	4,072	2,441	-1.5%	-11.9%	-36.0%	-22.3%	0.986 [0.961–1.013]	0.883^a^ [0.824–0.946]	0.654 ^a^ [0.629–0.679]	0.782 ^a^ [0.742–0.824]
**Surgery**	4,112	647	1,775	1,122	-5.9%	-7.3%	-36.0%	-19.4%	0.943 ^a^ [0.905–0.984]	0.927 [0.834–1.032]	0.647 ^a^ [0.610–0.686]	0.808 ^a^ [0.747–0.874]
**Intensive Care Unit**	709	100	286	186	2.3%	-21.3%	-36.4%	-18.4%	1.023 [0.922–1.135]	0.788 [0.606–1.023]	0.637 ^a^ [0.549–0.738]	0.816 ^a^ [0.673–0.990]

^a^ p<0.05

### Mortality

The trend of all-cause mortality was similar in 2019 and 2020, with a slight decline after the winter season (Fig 1c).

Joinpoint analysis revealed a significant difference between the two regression curves, with only one joinpoint in each: while deaths increased during the flu period up to week 2 in 2019 and then declined afterwards, in 2020 deaths were stable until week 15 and then declined significantly.

A small increase in all-cause overall mortality (7.5%) was observed only during lockdown (IRR 1.074, p<0.05, 95%CI [1.007–1.145]) (Table 2), whereas in both pre- and post-lockdown periods no significant change in mortality was detectable. Lockdown was associated with a significantly higher cause-specific mortality related to endocrine, nutritional and metabolic diseases (47.9%, IRR 1.478, p<0.05, 95%CI [1.094–1.998]) and diseases of the circulatory system (15.5%, IRR 1.154, p<0.05, 95%CI [1.031–1.291]). Conversely, mortality reduced only for infectious and parasitic diseases (-36.2%, IRR 0.638, p<0.05, 95%CI [0.437–0.931]). As for mental, behavioral and neuro-developmental disorders, a significant increase in mortality during the pre-lockdown period was observed, while deaths for neoplasms decreased during the post-lockdown weeks (Table 2).

**Table 2:** Number and percentage change of all-cause mortality (overall and cause-specific), in-hospital and out-of-hospital mortality (overall and cause-specific), year 2020 compared to 2019.

	2020 (n)				Change from previous year (%)				Incidence Rate Ratio [95% CI]			
	Flu Period	Pre-lockdown	Lock-down	Post-lockdown	Flu Period	Pre-lockdown	Lock-down	Post-lockdown	Flu Period	Pre-lockdown	Lock-down	Post-lockdown
**Certain infectious and parasitic diseases**	90	15	44	32	-12.6%	-25.0%	-36.2%	-3.0%	0.874 [0.659–1.159]	0.750 [0.384–1.465]	0.638 ^a^ [0.437–0.931]	0.970 [0.596–1.577]
**Neoplasms**	722	137	489	213	-2.2%	14.2%	2.1%	-22.0%	0.979 [0.883–1.084]	1.142 [0.894–1.458]	1.021 [0.900–1.158]	0.781 ^a^ [0.653–0.934]
**Endocrine, nutritional and metabolic diseases**	138	18	105	29	0.0%	20.0%	47.9%	-9.4%	1.000 [0.790–1.266]	1.200 [0.605–2.381]	1.478 ^a^ [1.094–1.998]	0.906 [0.548–1.498]
**Mental, Behavioral and Neurodevelopmental disorders**	131	35	83	45	-12.7%	84.2%	-3.5%	9.8%	0.874 [0.691–1.104]	1.842 ^a^ [1.054–3.219]	0.965 [0.714–1.305]	1.098 [0.719–1.676]
**Diseases of the nervous system**	118	23	72	24	18.0%	-8.0%	5.9%	-4.0%	1.180 [0.904–1.540]	0.920 [0.522–1.621]	1.059 [0.760–1.475]	0.960 [0.548–1.681]
**Diseases of the circulatory system**	915	161	650	229	-14.2%	-4.7%	15.5%	-16.7%	0.860 ^a^ [0.787–0.939]	0.953 [0.768–1.182]	1.154 ^a^ [1.031–1.291]	0.833 ^a^ [0.699–0.993]
**Diseases of the respiratory system**	325	49	218	60	-11.2%	-29.0%	19.8%	-26.8%	0.888 [0.765–1.031]	0.710 [0.493–1.024]	1.197 [0.984–1.458]	0.732 [0.525–1.021]
**Diseases of the digestive system**	100	14	64	28	-11.5%	0.0%	12.3%	0.0%	0.885 [0.676–1.158]	1.000 [0.477–2.098]	1.123 [0.786–1.604]	1.000 [0.592–1.688]
**Diseases of the genitourinary system**	62	19	36	24	-8.8%	58.3%	-25.0%	0.0%	0.912 [0.646–1.286]	1.583 [0.769–3.261]	0.750 [0.487–1.155]	1.000 [0.568–1.761]
**Injury, poisoning and certain other consequences of external causes**	120	24	77	35	-8.4%	9.1%	-2.5%	-16.7%	0.916 [0.715–1.173]	1.091 [0.612–1.945]	0.975 [0.712–1.334]	0.833 [0.532–1.305]
**Other** ^a^	103	16	55	21	21.2%	-5.9%	-6.8%	-34.4%	1.212 [0.909–1.614]	0.941 [0.476–1.863]	0.932 [0.646–1.346]	0.656 [0.379–1.138]
**All-cause overall mortality**	2,824	511	1,893	740	-7.7%	1.8%	7.5%	-16.6%	0.926 ^a^ [0.880–0.973]	1.018 [0.900–1.151]	1.074 ^a^ [1.007–1.145]	0.836 ^a^ [0.758–0.921]
**In-hospital overall mortality**	1,648	289	918	426	-6.5%	-4.9%	-15.0%	-19.3%	0.936 [0.876–1.000]	0.951 [0.810–1.117]	0.759 ^a^ [0.694–0.831]	0.808 ^a^ [0.711–0.917]
**Out-of-hospital overall mortality**	1,176	222	975	314	-9.2%	12.1%	43.2%	-12.5%	0.909 ^a^ [0.841–0.983]	1.121 [0.926–1.357]	1.428 ^a^ [1.295 a 1.574]	0.875 [0.752–1.018]
**Certain infectious and parasitic diseases**	15	1	7	3	66.7%	0.0%	133.3%	-25.0%	1.667 [0.729–3.808]	1.000 [0.063–15.988]	2.333 [0.603–9.023]	0.750 [0.168–3.351]
**Neoplasms**	232	42	228	85	-2.1%	50.0%	76.7%	-8.6%	0.979 [0.817–1.173]	1.500 [0.930–2.419]	1.766 ^a^ [1.4230–2.191]	0.914 [0.681–1.226]
**Endocrine, nutritional and metabolic diseases**	77	9	61	15	-2.5%	12.5%	79.4%	-6.3%	0.975 [0.712–1.334]	1.125 [0.434–2.916]	1.794 ^a^ [1.179–2.728]	0.937 [0.464–1.896]
**Mental, Behavioral and Neurodevelopmental disorders**	68	18	52	29	-17.1%	80.0%	40.5%	123.1%	0.829 [0.601–1.144]	1.780 [0.831–3.899]	1.405 [0.922–2.142]	2.230 ^a^ [1.160–4.290]
**Diseases of the nervous system**	73	14	52	12	40.4%	16.7%	36.8%	-14.3%	1.404 [0.984–2.003]	1.167 [0.540–2.522]	1.368 [0.901–2.079]	0.857 [0.397–1.853]
**Diseases of the circulatory system**	486	95	418	115	-15.9%	1.1%	32.7%	-21.2%	0.842 ^a^ [0.746–0.949]	1.011 [0.760–1.344]	1.326 ^a^ [1.146–1.534]	0.788 [0.617–1.006]
**Diseases of the respiratory system**	85	9	59	17	-13.3%	-43.8%	47.5%	-15.0%	0.868 [0.649–1.160]	0.563 [0.249–1.273]	1.477 [0.987–2.203]	0.850 [0.445–1.623]
**Diseases of the digestive system**	14	3	14	5	7.7%	-25.0%	40.0%	25.0%	1.077 [0.506–2.291]	0.750 [0.168–3.351]	1.400 [0.622–3.152]	1.250 [0.336–4.655]
**Diseases of the genitourinary system**	16	4	4	5	-20.0%	33.3%	-71.4%	-37.5%	0.800 [0.415–1.544]	1.333 [0.298–5.957]	0.286 ^a^ [0.094–0.868]	0.625 [0.205–1.911]
**Injury, poisoning and certain other consequences of external causes**	52	16	39	17	-27.8%	60.0%	11.4%	-15.0%	0.722 [0.506–1.032]	1.600 [0.726–3.525]	1.114 [0.706–1.759]	0.850 [0.445–1.623]
**Other** ^b^	58	11	41	11	5.5%	-8.3%	57.7%	-47.6%	1.055 [0.729–1.525]	0.917 [0.405–2.077]	1.577 [0.965–2.577]	0.524 [0.253–1.087]

^a^ p<0.05

^**b**^ Causes of deaths that accounted for ≤0.5% of the total (ICD-10-CM categories D50-89, H00-95, L00-99, M00-99, O00-9A, P00-96, Q00-99, R00-99, V00-Y99, Z00-99) were included in Other.

We found a significant increase in out-of-hospital mortality, particularly during the lockdown weeks (43.2%, IRR 1.428, p<0.05, 95%CI [1.295–1.574]), whereas in the pre-lockdown period and in the post-lockdown period no remarkable changes were identified. A concurrent reduction of in-hospital mortality was observed (-15.0%, IRR 0.759, p<0.05, 95%CI [0.694–0.831].

Analysis of cause-specific out-of-hospital mortality (Table 2) confirmed an increase in deaths for neoplasms, endocrine, nutritional and metabolic diseases, and diseases of the circulatory systems during lockdown. Conversely, we found a significant reduction only in genitourinary disorders.

## Discussion

Globally, Italy has been the first Western country to experience the disruptive effect of the pandemic in terms of cases, deaths and the related burden on healthcare services [1–3, 14]. Since the beginning of the pandemic, a reduction of ED visits and hospitalizations overall [6] and for specific diseases [14–18] have been reported worldwide.

In this paper we studied the pandemic consequences on non-COVID-19 patients in an area with over one million inhabitants. We highlighted how ED visits and hospitalizations reduced overall, and particularly during the lockdown period. To the best of our knowledge, this is the first study to analyze also the trends of in- and out-of-hospital, all-cause and cause-specific mortality, providing a comprehensive picture of the short-term consequences of the pandemic on non-COVID-19 patients.

Our findings indicate that the reduction of ED visits and hospitalizations started two weeks before the beginning of the national lockdown, after school closures and the first Italian case hospitalized for COVID-19 in Codogno [1], when no cases of local transmission had been still recorded in BMA. As already reported [2, 6, 19–23], a possible explanation is that the population response is likely to be more affected by the national level authority risk message than the real local situation. Several possible reasons have been put forward to explain, at least partially, a reduction in ED visits and hospitalizations [6, 7], such as lifestyle changes or fear of the contagion, as well as the increasingly stringent lockdown measures and the postponement of elective procedures, or the sense of civic responsibility of the population [7].

It should be highlighted that not all ED visits represent real emergencies, and inappropriate accesses to ED are a well-known issue related to several reasons [7, 24–26]. That being said, the observed reduction of the less severe triage codes was to somewhat expected, although not to this large extent. What we were not prepared for was the concurrent decrease of the most serious cases. Furthermore, we showed how ED visits’ reduction encompassed all pathological conditions, thus including also acute and time-dependent diseases, similarly to what already reported in literature [27–29]. The overall reduction of ED visits, regardless of age, severity, and causes, suggests the system’s inability to guide the patient in discriminating the real need for urgent care. Therefore, public awareness should be taken into due consideration to be prepared for a recrudescence of the pandemic.

A clear reduction of non-COVID-19 patients hospitalization has also been recorded, both for medical and surgical diseases and for conditions requiring intensive care support. Data on hospital admissions reported in previous studies [6, 16] appear to be not comparable, as obtained from selected populations [16] or different healthcare systems, such as U.S. care system or other insurance-based systems [6]. In fact, unlike the U.S., the Italian National Healthcare Service is a single-payer system funded by general taxation, similarly to the UK national health service [30]. This could have an impact on a system’s hospitalization rate [31].

Looking at the post-lockdown period, when the spread of COVID-19 was contained, we expected to see a progressive increase in ED visits and a consequent increase in hospitalizations, as a consequence of the delay in obtaining medical attention and care access due to lockdown. However, even if these indicators started to rise during the lockdown weeks and throughout the post-lockdown period, they did not reach the pre-pandemic levels.

The all-cause mortality showed only a minimal increase (+7.5%) during the lockdown period. The significant increase in mortality for cardiovascular and endocrine systems’ diseases that we have registered during lockdown is in line with other studies [27–29, 31, 32].

The key finding of our study is that, with the sudden drop of ED visits and hospitalizations, we found a statistically significant increase of out-of-hospital all-cause mortality, mainly driven by an increase in deaths for neoplasms, cardiovascular diseases and endocrine, nutritional and metabolic diseases. In-hospital mortality instead showed an opposite and decreasing trend, due to the reduction of non-COVID-19 patients in healthcare facilities. We assume that these data are attributable to a delay in seeking hospital care and to system’s inability in providing the same standards of care during a public health emergency, both in terms of quality and quantity.

During the pre-lockdown weeks, we observed a significant increase in mortality for mental diseases. This might be due to the sense of fear and anxiety which reigned during the pre-lockdown period, with a consequent exacerbation of mental disorders [33, 34]. The reduction in overall mortality for neoplasms during the post-lockdown weeks, when considered together with the increased out-of-hospital mortality for the same cause recorded in the lockdown period, likely needs to be analyzed when medium- and long-term data will be available.

During the first COVID-19 wave the reduction in ED visits and hospitalization probably eased the healthcare system in facing an unprecedented emergency. In view of the recurrence of the crisis, the scenario described by our study represents a teachable moment about population and healthcare systems’ response at the early stages of the pandemic in terms of reduced demand of care and systems’ capability in intercepting it.

### Study limitations

This study has some limitations. First, mortality data might include under-diagnosed COVID-19 cases. In fact, the type of access and causes of death could have been coded incorrectly, due to the uncertain case definition and the context of that time-period, when the pandemic disruptively changed routine practice. Therefore, it is possible that non-COVID-19 mortality is overestimated. Furthermore, we had access to only to aggregated data on mortality data by age group, triage codes and cause, which prevented us from conducting age- and gender-adjusted analyses. Lastly, the short time window of the post-lockdown period considered in this study does not allow us to generalize our findings to possible medium- and long-term effects of the pandemic.

## Conclusions

In summary, our analysis showed a clear reduction of ED visits and hospitalizations for non-COVID-19 patients during the first months of the pandemic in a wide and populated area, and a significant increase in non-COVID-19 out-of-hospital deaths.

Out-of-hospital mortality due to neoplasms, cardiovascular and endocrine systems’ diseases showed a significant increase particularly during the lockdown weeks, while for other conditions the consequences of the pandemic on non-COVID-19 patients are still unknown.

According to our findings, we advocate the need for further investigations into the medium- and long-term effects of the pandemic on non-COVID-19 patients, in order to strengthen population awareness and healthcare systems’ preparedness.

## Supporting information

S1 File (DOCX)
