# Supplementary material for: Non-COVID-19 patients in times of pandemic: Emergency department visits, hospitalizations and cause-specific mortality in Northern Italy
Source: PLoS One. 2021 Mar 22;16(3):e0248995. doi: 10.1371/journal.pone.0248995 (PMC7984614; doi:10.1371/journal.pone.0248995)
Supplement: S1 File — (DOCX) [file pone.0248995.s001.docx]

**S1 Table.** Demographic and population characteristics.

|  | **2019** | | **2020** | |
| --- | --- | --- | --- | --- |
| **Age (n, mean, SD)** | 1,016,792 | (46.0 ± 23.7) | 1,019,875 | (46.1 ± 23.7) |
| **< 15 yrs (n, %)** | 130,468 | 12.8% | 128,934 | 12.6% |
| **15-64 yrs (n, %)** | 638,491 | 62.8% | 641,937 | 62.9% |
| **>=65 yrs (n, %)** | 247,833 | 24.4% | 249,004 | 24.4% |
| **Male (n, %)** | 490,325 | 48.2% | 492,154 | 48.3% |

SD: Standard Deviation

Source: <https://statistica.regione.emilia-romagna.it/servizi-online/statistica-self-service/popolazione/popolazione-residente-dal-1861>

**S2 Table.** ED visits and hospitalizations: absolute value and percentage change compared to the previous year.

| **ED visits** | **2019** | **2020** | **Change (%) from previous year** |
| --- | --- | --- | --- |
| flu-period | 109,169 | 109,365 | 0.2% |
| pre-lockdown | 19,134 | 11,821 | -38.2% |
| lockdown | 74,222 | 25,086 | -66.2% |
| post lockdown | 36,016 | 19,033 | -47.2% |
|  |  |  |  |
| **Hospitalizations** | **2019** | **2020** | **Change (%) from previous year** |
| flu-period | 16,837 | 16,620 | -1.3% |
| pre-lockdown | 2,813 | 2,192 | -22.1% |
| lockdown | 10,802 | 5,820 | -46.1% |
| post lockdown | 5,338 | 3,513 | -34.2 |

**S3 Table.** Percentage change in ED visits by age group compared to the previous year.

|  | | Dec2018-May2019 | 2019-2020 | | | |
| --- | --- | --- | --- | --- | --- | --- |
|  |  |  | ED visits w/o COVID-19 | | ED visits Total | |
|  |  |  | N | *change from previous year* | N | *change from previous year* |
| flu period | <15 | 18,693 | 18,265 | *-2.3%* | 18,270 | *-2.3%* |
|  | 15-64 | 58,080 | 58,843 | *1.3%* | 58,849 | *1.3%* |
|  | >= 65 | 32,016 | 32,644 | *2.0%* | 32,645 | *2.0%* |
| pre-lockdown | <15 | 3,142 | 1,443 | *-54.1%* | 1,445 | *-54.0%* |
|  | 15-64 | 10,376 | 7,956 | *-23.3%* | 8,008 | *-22.8%* |
|  | >= 65 | 5,399 | 3,935 | *-27.1%* | 3,983 | *-26.2%* |
| lockdown | <15 | 12,141 | 2,038 | *-83.2%* | 2,163 | *-82.2%* |
|  | 15-64 | 39,953 | 14,369 | *-64.0%* | 17,256 | *-56.8%* |
|  | >= 65 | 21,209 | 7,865 | *-62.9%* | 9,780 | *-53.9%* |
| post-lockdown | <15 | 5,352 | 1,804 | *-66.3%* | 1,874 | *-65.0%* |
|  | 15-64 | 19,959 | 10,802 | *-45.9%* | 11,344 | *-43.2%* |
|  | >= 65 | 10,345 | 6,076 | *-41.3%* | 6,578 | *-36.4%* |

**S4 Table.** Specific causes of ED visits: absolute value and percentage change (%) compared to the previous year ^a^. Note: [ICD-9-CM specific codes]

|  | | 2020 | | | | | |
| --- | --- | --- | --- | --- | --- | --- | --- |
|  |  | pre-lockdown | Change (%) from previous year | lockdown | Change (%) from previous year | post- lockdown | Change (%) from previous year |
| Infectious and parasitic diseases (001-139) | 1 Intestinal infections due to non specified organism [008.5 - 009.3] | 72 | *-67.0%* | 71 | *-92.5%* | 75 | *-74,5%* |
|  | 2 Erysipelas [035] | 21 | *-44.7%* | 40 | *-73.3%* | 32 | *-54,9%* |
|  | 3 Septicemia [038 - 038.9] | 6 | *20.0%* | 11 | *0.0%* | 6 | *-50,0%* |
|  | Other diseases * | 194 | *-31.0%* | 292 | *-77.8%* | 209 | *-67,1%* |
|  | Total | 293 | *-45.9%* | 414 | *-82.9%* | 322 | *-68,2%* |
| Neoplasms (140-239) | | 53 | *-7.0%* | 148 | *-40.8%* | 99 | *-15.4%* |
| Endocrine, nutritional and metabolic diseases, and immunity disorders (240-279) | 4 Diabetes, manifestations and complications [250 - 250.93] | 31 | *-18.4%* | 68 | *-55.8%* | 44 | *-36,2%* |
|  | 5 Hypoglicemia [251.0, 251.2] | 12 | *9.1%* | 15 | *-71.7%* | 12 | *20,0%* |
|  | 6 Disorders of fluid, electrolyte, and acid-base balance [276 - 276.9] | 25 | *-57.6%* | 57 | *-65.9%* | 46 | *-51,1%* |
|  | Other diseases* | 12 | *-42.9%* | 25 | *-62.7%* | 14 | *-60,0%* |
|  | Total | 80 | *-38.0%* | 165 | *-62.6%* | 116 | *-44,2%* |
| Diseases of the blood and blood-forming organs (280-289) | 7 Anemia [280.0 - 285.9] | 46 | *-29.2%* | 117 | *-53.9%* | 80 | *-41,2%* |
|  | Other diseases* | 13 | *-27.8%* | 15 | *-76.2%* | 10 | *-76,2%* |
|  | Total | 59 | *-28.9%* | 132 | *-58.4%* | 90 | *-49,4%* |
| Mental disorders (290-319) | 8 Organic psychotic conditions [290 - 294.9] | 29 | *-31.0%* | 99 | *-47.1%* | 66 | *-30,5%* |
|  | Other diseases* | 918 | *-7.8%* | 1895 | *-54.6%* | 1356 | *-37,8%* |
|  | Total | 947 | *-8.8%* | 1994 | *-54.3%* | 1422 | *-37,5%* |
| Diseases of the nervous system and sense organs (320-389) | 9 Inflammatory diseases of the central nervous system [320 - 324.9] | 1 | *-66.7%* | 2 | *-77.8%* | 3 | *50,0%* |
|  | 10 Hereditary and degenerative diseases of the central nervous system [330.0 - 337.9] | 7 | *-50.0%* | 12 | *-77.4%* | 17 | *-26,1%* |
|  | 11 Pain [338.0 - 338.4] | 114 | *-40.9%* | 187 | *-71.3%* | 121 | *-69,3%* |
|  | 12 Epilepsy and recurrent seizures [345 - 345.91] | 48 | *2.1%* | 91 | *-52.4%* | 66 | *-25,8%* |
|  | 13 Retinal disorders [361 - 362.89] | 36 | *-21.7%* | 79 | *-48.7%* | 51 | *-50,0%* |
|  | 14 Vertiginous syndromes and other disorders of vestibular system [386.1 - 386.2] | 79 | *-28.8%* | 110 | *-79.0%* | 115 | *-55,6%* |
|  | Other diseases* | 831 | *-35.7%* | 1251 | *-76.4%* | 1124 | *-56,0%* |
|  | Total | 1116 | *-34.6%* | 1732 | *-74.8%* | 1497 | *-56,3%* |
| Diseases of the circulatory system (390-459) | 15 Hypertensive disease [401 - 405.99] | 96 | *-34.2%* | 173 | *-65.4%* | 119 | *-51,0%* |
|  | 16 Ischemic heart disease [410 - 414.9] | 55 | *-27.6%* | 153 | *-49.7%* | 94 | *-39,7%* |
|  | 17 Acute pulmonary heart disease [415.0 - 415.19] | 9 | *0.0%* | 26 | *-31.6%* | 24 | *0,0%* |
|  | 18 Acute pericarditis [420 - 420.99] | 2 | *-33.3%* | 16 | *-23.8%* | 5 | *-61,5%* |
|  | 19 Acute myocarditis [422 - 422.99] | 1 | *0.0%* | 2 | *-50.0%* | 2 | *100,0%* |
|  | 20 Conduction disorders and dysrhythmias [426 - 427.9] | 151 | *-29.1%* | 352 | *-58.6%* | 263 | *-33,9%* |
|  | 21 Heart failure [428 - 428.9] | 123 | *-29.7%* | 263 | *-59.8%* | 200 | *-25,4%* |
|  | 22 Intracranial hemorrhages [430 - 432.9] | 20 | *-20.0%* | 72 | *-33.3%* | 45 | *-6,3%* |
|  | 23 Ischemic cerebral disorders [433.0 – 435.9] | 111 | *11.0%* | 221 | *-45.8%* | 145 | *-27,5%* |
|  | 24 Aortic aneurysm and dissection [441 – 441.9] | 6 | *20.0%* | 21 | *-22.2%* | 5 | *-37,5%* |
|  | Other diseases* | 121 | *-44.5%* | 230 | *-67.8%* | 206 | *-41,5%* |
|  | Total | 695 | *-28.4%* | 1529 | *-57.9%* | 1108 | *-35,2%* |
| Diseases of the respiratory system (460-519) | 25 Upper respiratory tract infections [460 - 478.9] | 501 | *-34.8%* | 389 | *-83.9%* | 155 | *-83,3%* |
|  | 26 Pneumonia and influenza [480.8 – 487.8] | 400 | *1.0%* | 391 | *-59.0%* | 88 | *-79,1%* |
|  | 27 Chronic obstructive pulmonary disease and allied conditions [490 – 495.9] | 108 | *-30.8%* | 112 | *-76.6%* | 77 | *-67,8%* |
|  | 28 Pleurisy [511.0 – 511.9] | 16 | *-20.0%* | 50 | *-47.4%* | 31 | *-32,6%* |
|  | Other diseases* | 134 | *-22.5%* | 212 | *-59.2%* | 82 | *-65,1%* |
|  | Total | 1159 | *-23.4%* | 1154 | *-74.1%* | 433 | *-76,9%* |
| Diseases of the digestive system (520-579) | 29 Diseases of esophagus, stomach, and duodenum [530.0 – 537.9] | 118 | *-43.8%* | 236 | *-68.5%* | 170 | *-51,0%* |
|  | 30 Appendicitis [540 – 543.9] | 29 | *-50.8%* | 128 | *-24.3%* | 84 | *-36,8%* |
|  | 31 Hernia of abdominal cavity [550 – 553.9] | 19 | *-24.0%* | 57 | *-58.1%* | 44 | *-32,3%* |
|  | 32 Noninfectious enteritis and colitis [555 – 558.9] | 37 | *-43.9%* | 49 | *-81.1%* | 35 | *-66,7%* |
|  | 33 Intestinal obstruction without mention of hernia [560 – 560.9] | 56 | *1.8%* | 119 | *-41.7%* | 84 | *-33,3%* |
|  | 34 Diverticula of intestine [562 – 562.12] | 24 | *-31.4%* | 45 | *-65.4%* | 35 | *-52,7%* |
|  | 35 Liver disease [570 – 573.9] | 12 | *-47.8%* | 33 | *-53.5%* | 21 | *-47,5%* |
|  | 36 Gall bladder and biliary tract disease [574 – 576.9] | 46 | *0.0%* | 129 | *-50.6%* | 87 | *-43,9%* |
|  | 37 Disease of pancreas [577.0 – 577.9] | 18 | *38.5%* | 48 | *-25.0%* | 23 | *-42,5%* |
|  | 38 Gastrointestinal hemorrhage [578 – 578.9] | 71 | *16.4%* | 141 | *-35.6%* | 59 | *-53,2%* |
|  | Other diseases* | 106 | *-51.6%* | 269 | *-66.2%* | 191 | *-57,2%* |
|  | Total | 536 | *-34.0%* | 1254 | *-59.0%* | 833 | *-49,7%* |
| Diseases of the genitourinary system (580-629) | 39 Renal failure [584 – 586] | 32 | *28.0%* | 59 | *-43.8%* | 47 | *-7,8%* |
|  | 40 Infections of kidney [590 – 590.9] | 14 | *0.0%* | 30 | *-52.4%* | 22 | *-42,1%* |
|  | 41 Hydonephrosis and kidney and ureter calculus [591 – 592.9] | 6 | *-62.5%* | 24 | *-44.2%* | 14 | *-48,1%* |
|  | 42 Cystitis and Urinary tract infection, site not specified [595 – 595.9, 599.0] | 101 | *-15.1%* | 152 | *-73.5%* | 126 | *-49,8%* |
|  | 43 Hematuria [599.7] | 51 | *-35.4%* | 103 | *-56.4%* | 75 | *-40,0%* |
|  | 44 Torsion of testis [608.2 – 608.20] | 3 | *-25.0%* | 9 | *-25.0%* | 8 | *100,0%* |
|  | 45 Disease of female pelvic organs and genital tract [614 – 629.9] | 106 | *-44.2%* | 264 | *-62.7%* | 187 | *-49,2%* |
|  | Other diseases* | 85 | *6.3%* | 144 | *-54.6%* | 81 | *-48,7%* |
|  | Total | 398 | *-24.5%* | 785 | *-61.9%* | 560 | *-45,2%* |
| Complications of pregnancy, childbirth, and the puerperium (630-677) | 46 Ectopic and molar pregnancy [632 – 633.91] | 19 | *5.6%* | 63 | *-25.0%* | 35 | *-41,7%* |
|  | 47 Other pregnancy with abortive outcome [634 – 639.9] | 47 | *23.7%* | 96 | *-29.4%* | 47 | *-53,0%* |
|  | 48 Complications mainly related to pregnancy [640.0 – 649.44] | 159 | *25.2%* | 354 | *-39.9%* | 233 | *-23,6%* |
|  | 49 Normal delivery, and other indications for care in pregnancy, labor, and delivery [650 – 659.93] | 102 | *-8.9%* | 318 | *-30.7%* | 180 | *-25,9%* |
|  | Other diseases* | 31 | *14.8%* | 39 | *-57.1%* | 33 | *-26,7%* |
|  | Total | 358 | *11.2%* | 870 | *-36.0%* | 528 | *-29,9%* |
| Diseases of the skin and subcutaneous tissue (680-709) | | 247 | *-39.8%* | 337 | *-80.4%* | 360 | *-59.0%* |
| Diseases of the musculoskeletal system and connective tissue (710-739) | 50 Arthropathies and related disorders [710 – 719.99] | 368 | *-41.1%* | 643 | *-71.4%* | 612 | *-48,7%* |
|  | 51 Dorsopathies [720 – 724.9] | 406 | *-37.5%* | 510 | *-79.8%* | 555 | *-56,9%* |
|  | 52 Rheumatism, excluding the back [725 – 729.9] | 162 | *-33.9%* | 243 | *-74.0%* | 224 | *-52,1%* |
|  | Other diseases* | 16 | *-38.5%* | 46 | *-66.7%* | 47 | *-25,4%* |
|  | Total | 952 | *-38.4%* | 1442 | *-75.4%* | 1438 | *-52,3%* |
| Congenital anomalies (740-759) | | 37 | *-66.7%* | 73 | *-78.3%* | 47 | *-45.3%* |
| Certain conditions originating in the perinatal period (776-779) | | 58 | *0.0%* | 175 | *-34.0%* | 152 | *17.8%* |
| Symptoms, signs, and ill-defined conditions (780-799) | 53 General symptoms [780 – 780.99] | 570 | *-2.2%* | 1034 | *-50.1%* | 608 | *-40,9%* |
|  | 54 Symptoms involving skin and other integumentary tissue [782.0 – 782.9] | 35 | *-30.0%* | 76 | *-66.8%* | 42 | *-60,7%* |
|  | 55 Symptoms involving head and neck [784.0 – 784.9] | 158 | *-39.0%* | 325 | *-65.3%* | 189 | *-58,6%* |
|  | 56 Symptoms involving cardiovascular system [785.0 – 785.9] | 84 | *1.2%* | 135 | *-58.3%* | 101 | *-47,7%* |
|  | 57 Symptoms involving respiratory system and other chest symptoms [786.0 – 786.9] | 476 | *-21.6%* | 1149 | *-49.2%* | 623 | *-43,8%* |
|  | 58 Symptoms involving digestive system [787.0 – 787.99] | 88 | *-53.7%* | 153 | *-76.7%* | 118 | *-51,6%* |
|  | 59 Symptoms involving urinary system [788.0 – 788.9] | 248 | *-13.3%* | 520 | *-53.9%* | 382 | *-36,1%* |
|  | 60 Other symptoms involving abdomen and pelvis [789 – 789.9] | 319 | *-37.5%* | 547 | *-74.1%* | 460 | *-57,4%* |
|  | Other diseases* | 26 | *-10.3%* | 48 | *-59.7%* | 33 | *-48,4%* |
|  | Total | 2004 | *-22.8%* | 3987 | *-59.5%* | 2556 | *-47,6%* |
| Injury and poisoning (800-999) | 61 Traumatisms of the locomotor apparatus [800 – 848.9] | 939 | *-24.6%* | 1733 | *-66.5%* | 1680 | *-32,1%* |
|  | 62 Intracranial injury, excluding those with skull fracture [850 – 854.19] | 208 | *-11.5%* | 375 | *-62.6%* | 301 | *-36,9%* |
|  | 63 Internal injury of thorax, abdomen, and pelvis [860 – 869.1] | 6 | *-45.5%* | 25 | *-39.0%* | 27 | *50,0%* |
|  | 64 Sepsis and severe sepsis [995.91 – 995.92] | 43 | *-29.5%* | 131 | *-44.0%* | 101 | *-9,8%* |
|  | Other diseases* | 1810 | *-36.3%* | 2774 | *-75.2%* | 2905 | *-46,7%* |
|  | Total | 3006 | *-31.6%* | 5038 | *-71.4%* | 5014 | *-41,2%* |
| External causes of injury and supplemental classification (codes V) | | 632 | *-18.3%* | 2581 | *-14.9%* | 1486 | *0.7%* |

^a^ No remarkable changes of ED visits by disease category have been found during the flu period.
* Other diseases include all the codes not listed elsewhere within the disease category.

**S1 Fig.** Joinpoint analysis of ED visits


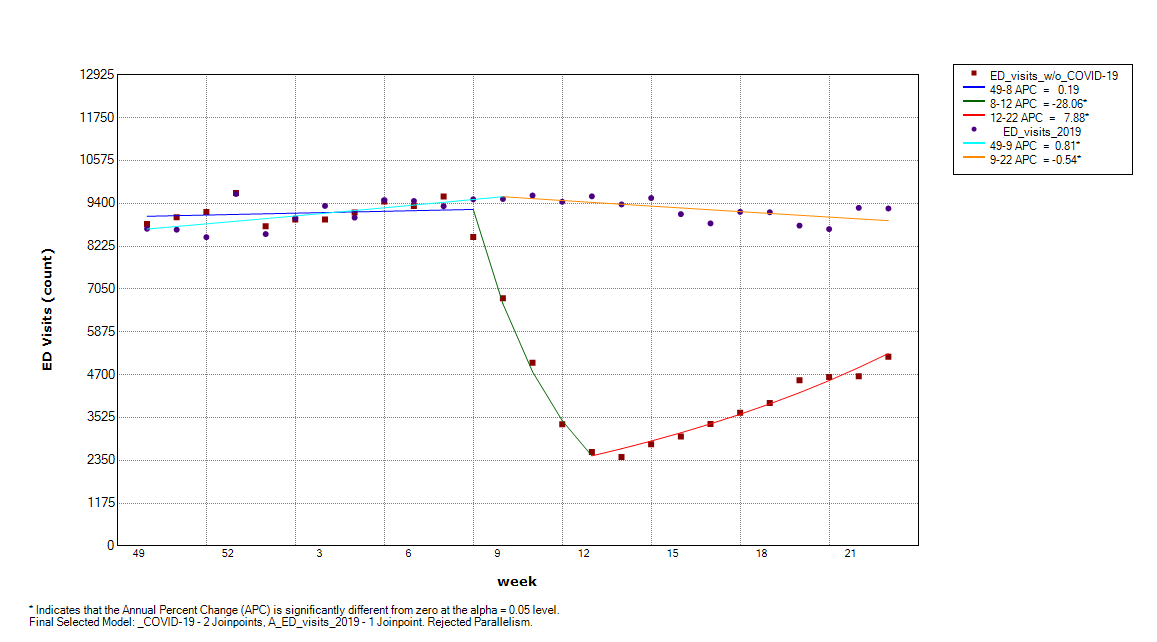


**S2 Fig.** Joinpoint analysis of hospitalizations


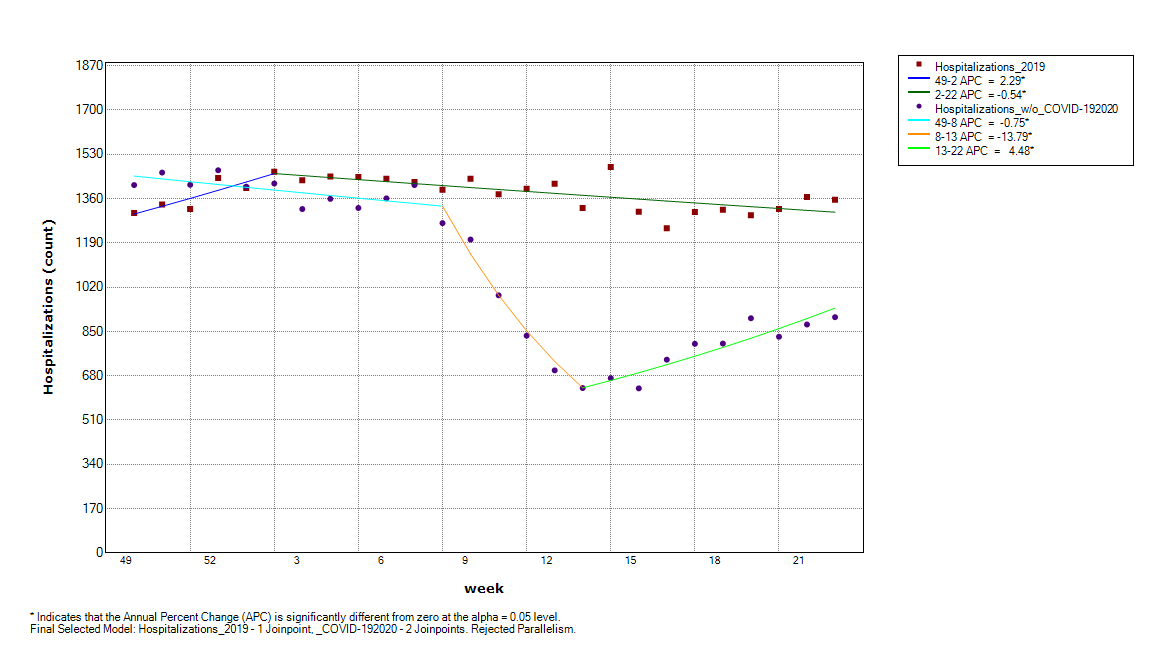


**S3 Fig.** Joinpoint analysis of deaths.


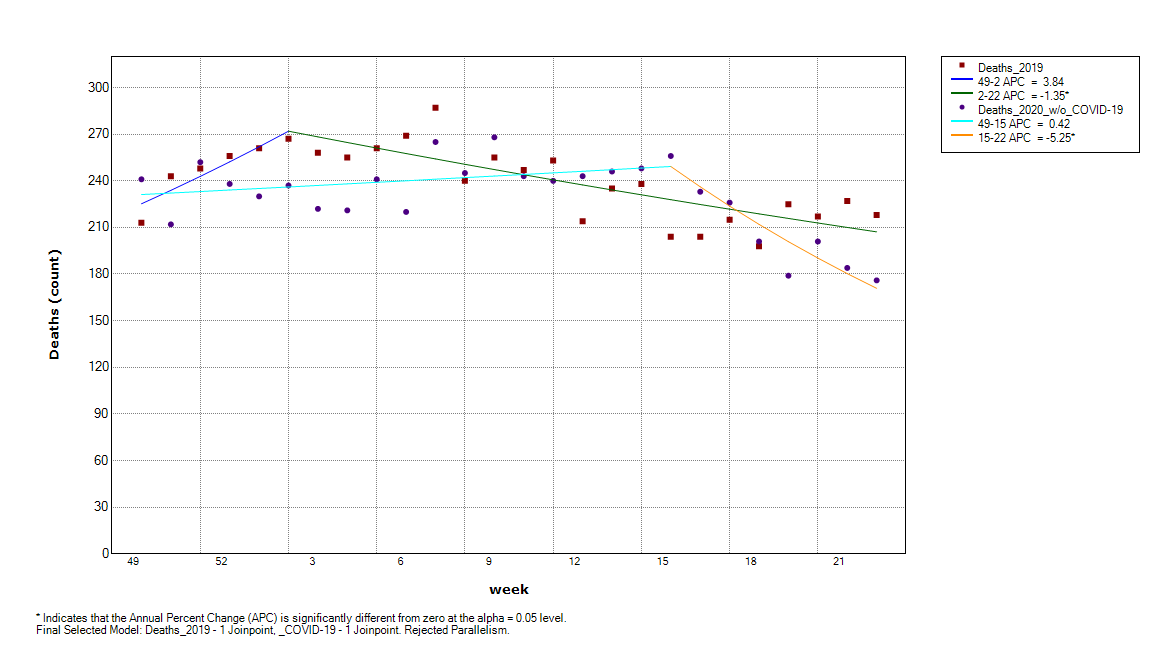


**Algorithms and ICD-9/ICD-10 codes to identify COVID-19 cases and diagnostic groups**

**ED-visits and hospitalizations (ICD-9-CM codes)**

001-139: Infectious and parasitic diseases

140-239: Neoplasms

240-279: Endocrine, nutritional and metabolic diseases, and immunity disorders

280-289: Diseases of the blood and blood-forming organs

290-319: Mental disorders

320-389: Diseases of the nervous system and sense organs

390-459: Diseases of the circulatory system

460-519: Diseases of the respiratory system

520-579: Diseases of the digestive system

580-629: Diseases of the genitourinary system

630-677: Complications of pregnancy, childbirth, and the puerperium

680-709: Diseases of the skin and subcutaneous tissue

710-739: Diseases of the musculoskeletal system and connective tissue

740-759: Congenital anomalies

776-779: Certain conditions originating in the perinatal period

780-799: Symptoms, signs, and ill-defined conditions

800-999: Injury and poisoning

Codes V: External causes of injury and supplemental classification.

On March 10, 2020, with decision PG/2020/210759, the Emilia-Romagna Regional Agency for healthcare Services issued the criteria to identify and record patients with COVID-19 disease in the administrative databases. The criteria are also reported in the attached document (GUIDELINES FOR THE CODIFICATION OF CASES WITH SARS-COV-2 (COVID-19) DISEASE, Supporting Information 2) from the Italian Ministry of Health, released on March 26, 2020. We report a Table with the criteria extracted from the original document.

Table. COVID-19 related ICD-9-CM codes

| **Patient condition** | **ICD-9-CM codes** |
| --- | --- |
| Patient confirmed contact with a coronavirus positive case | V01.82 “Exposure to SARS-Coronavirus associated” |
| Patient without pneumonia | 079.82 “SARS-Coronavirus associated” |
| Patient with pneumonia | 480.3 “SARS-Coronavirus pneumonia associated” |
| Need for confinement | V07.0 |

**Mortality (ICD-10-CM codes)**

A00-B99: Certain infectious and parasitic diseases

C00-D48: Neoplasms

D50-D53: Diseases of the blood and blood-forming organs

E00-E90: Endocrine, nutritional and metabolic diseases, and immunity disorders

F00-F99: Mental disorders

G00-G99: Diseases of the nervous system and sense organs

I00-I99: Diseases of the circulatory system

J00-J99: Diseases of the respiratory system

K00-K99: Diseases of the digestive system

L00-L99: Diseases of the skin and subcutaneous tissue

M00-M99: Diseases of the musculoskeletal system and connective tissue

N00-N99: Diseases of the genitourinary system

O00-O99: Complications of pregnancy, childbirth, and the puerperium

S00-T98: Injury and poisoning

U07: COVID-19
